# Conservative vs. Surgical Management for Femoro-Acetabular Impingement: A Systematic Review of Clinical Evidence

**DOI:** 10.3390/jcm11195852

**Published:** 2022-10-02

**Authors:** Giuseppe Anzillotti, Alberto Iacomella, Matteo Grancagnolo, Enrico Maria Bertolino, Maurilio Marcacci, Cristiano Sconza, Elizaveta Kon, Berardo Di Matteo

**Affiliations:** 1 Department of Biomedical Sciences, Humanitas University, 20072 Pieve Emanuele, MI, Italy; 2 IRCCS Humanitas Research Hospital, 20089 Rozzano, MI, Italy

**Keywords:** femoro-acetabular impingement, hip, arthroscopy, cam, pincer, physical therapy

## Abstract

Femoro-acetabular impingement (FAI) syndrome is one of the most studied conditions in sports medicine. Surgical or conservative approaches can be proposed for treating FAI, although the best standard of care is not established yet. Our aim is to provide a comprehensive review of the best treatment for FAI syndrome evaluating differences in outcomes between surgical and non-operative management. A literature search was carried out on the PubMed, EMBASE, Scopus, and PEDro databases, using the following keywords: “femoroacetabular impingement”, “FAI”, in association with “surgery”, “arthroscopy”, “surgical” and “conservative”, “physiotherapy”, “physical therapy”, “rehabilitation”, “exercise”. Only Level I RCTs were included. Four articles were selected for this systematic review. Our analysis showed different therapeutic protocols, follow-up periods, and outcomes; however, three out of the four studies included favored surgery. Our study demonstrates beneficial effects for both arthroscopic treatment and a proper regimen of physical therapy, nevertheless a surgical approach seemed to offer superior short-term results when compared to conservative care only. Further trials with larger sample sizes and longer follow-ups are needed to assess the definitive approach to the FAI condition.

## 1. Introduction

Femoro-acetabular impingement (FAI) syndrome is defined as the triad of symptoms, clinical signs and imaging findings in which structural morphology results in conflict between the femoral head and the acetabulum [1]. First described during the 1990s [2,3,4], the incidence of FAI morphology, which could induce the syndrome, is estimated to be up to 37% and 67% in asymptomatic patients for cam and pincer, respectively [5]. Although FAI syndrome is one of the most studied conditions in sports medicine, its etiology still remains unknown [6]. 

Abnormal sphericity of the femoral head (cam or pistol grip), excessive protrusion of acetabular edge (pincer), or both, may lead to mechanical conflict leading to the syndrome [7].

**Figure 1:**
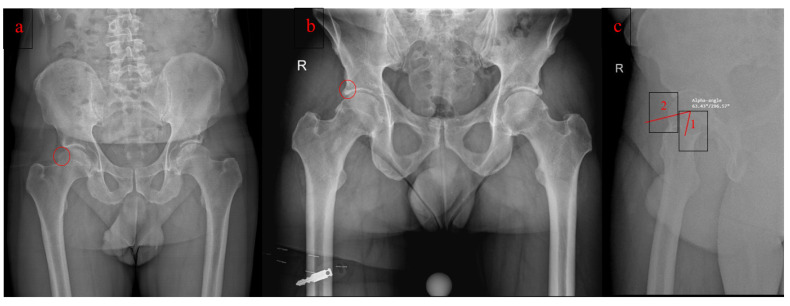
(**a**). FAI Cam type (**b**). FAI Pincer type (**c**). α angle in a lateral view: line 1 passing from the center of the femoral head to the center of the femoral neck and line 2 passing from the center of the femoral head to a point where the distance from the bone to the center of the head is greater than the radius of the cartilage covered femoral head.

Cam-type (Figure 1a) morphology results in impingement due to an abnormal-shaped femoral head that rotates into the acetabulum, especially during forceful flexion. Repetitive end-of-motion movements result in shearing and disruption of the acetabular cartilage from the labrum.

Pincer-type (Figure 1b) morphology is based on an abnormal acetabular component that overextends and can be localized (acetabular retroversion) or involve the whole acetabulum (coxa profunda or protrusio acetabuli). The pincer type is also characterized by labral degeneration caused by repeated impingement.

Subjective symptoms, and clinical and radiologic findings are the fundamental pillars to diagnose FAI syndrome. Clinical examination reveals pain in the hip region represented by the “C sign”, decreased range of motion, and positivity to provocative tests (FADDIR and FABER) [8]. Radiologic findings are focused on X-ray measurements of the α angle (Figure 1c) for cam-type [1] (the angle between a line passing from the center of the femoral head to the center of the femoral neck and a second line passing from the center of the femoral head to a point where the distance from the bone to the center of the head is greater than the radius of the cartilage covered femoral head) [9] and detection of retroversion or over-coverage for pincer type [1,10].

Either surgical or conservative approaches can be proposed for treating FAI morphologies [11].

Conservative treatment mainly consists of supervised physical therapy, primarily tailored to the individual patient’s needs and desired level of function. Commonly before starting the physical therapy, a detailed clinical examination is performed to assess the patient’s impairments and adjust the exercise regimen that will be administered. Pain, function, and range of motion are established during the clinical examination [12,13]. During this initial evaluation, the physician has to train the patient on the condition and its management, including pain relief advice. Milestones of the therapy include joint mobilization, therapeutic exercises, soft tissue mobility, stretching, and motor control exercises. Avoiding impingement positions is also suggested. The frequency and number of exercise sessions vary among different rehabilitative centers. The exercises are usually first performed under the guidance of an experienced physiotherapist and can then be continued either in rehabilitative centers or at home [14,15]. Surgical treatment is also tailored to the patient’s type of impingement and is performed arthroscopically [16,17].

Arthroscopic treatments adopted are acetabuloplasty, femoroplasty, labral repair or debridement, and treatment of articular cartilage and ligament teres lesions, depending on the type of impingement present and damage to the adjacent structures. To date, the standard of care for the treatment of FAI has not been encoded and appears urgent to better define the most congruous approach to this disease [18].

The aim of the present systematic review is to provide a comprehensive analysis of the treatment of FAI syndrome, focusing exclusively on all the published Level I evidence studies available to elucidate the difference in outcomes between surgical and non-operative management.

## 2. Materials and Methods

**Figure 2:**
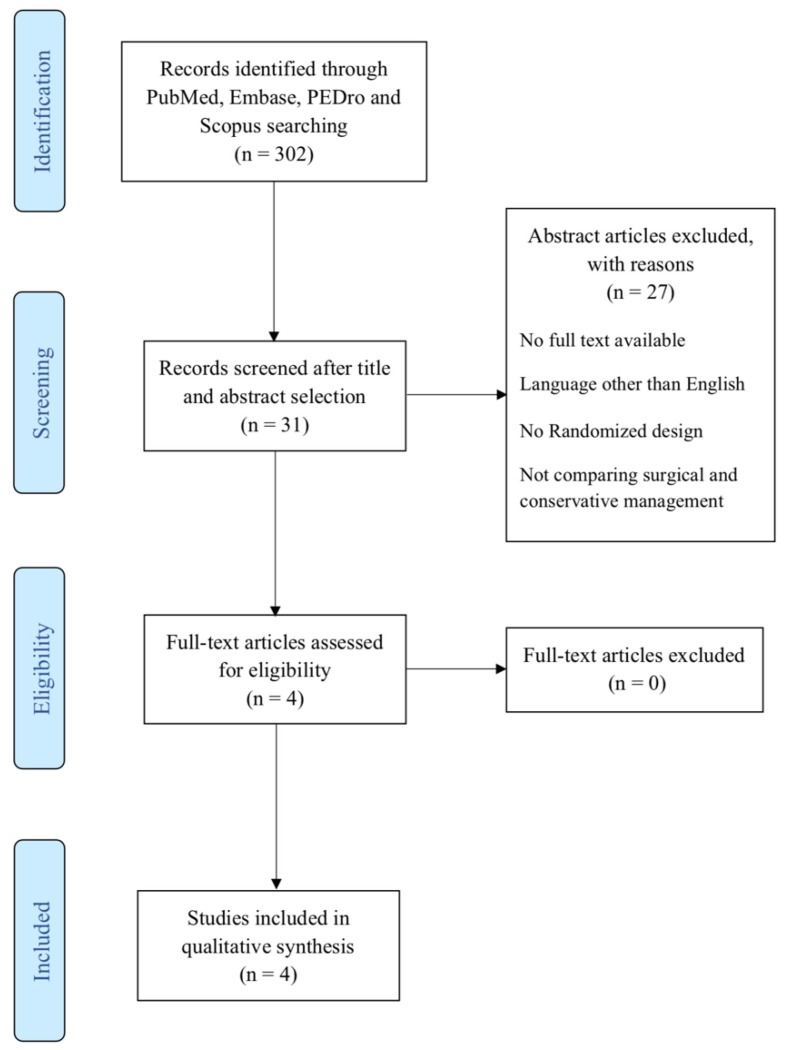
PRISMA Flowchart summarizing the selection process.

The present systematic review was performed according to “PRISMA guidelines” [Preferred Reporting Items for Systematic Reviews and Meta-analyses]. A literature search was carried out on the PubMed, EMBASE, Scopus, and PEDro databases, on June 30th, 2022, by two independent investigators, using the following keywords that were combined to achieve maximum search strategy sensitivity: “femoroacetabular impingement”, “FAI”, in association with: “surgery”, “arthroscopy”, “surgical” and “conservative”, “physiotherapy”, “physical therapy”, “rehabilitation”, “exercise”. Manual research throughout the reference lists of all retrieved articles was further conducted. A PRISMA flowchart of the selection and screening method is provided in Figure 2.

First, all the retrieved articles were screened by title and abstract, using the following inclusion criteria for article selection: (1) clinical reports with randomized design (level I) comparing conservative management to surgery; (2) written in the English language; (3) published from 2000 to 2022; (4) dealing with the treatment of patients affected by FAI syndrome. “Treatment” meant both surgery and conservative management, including exercise therapy, physical therapy (e.g., laser therapy, ultrasounds, shockwave therapy) and injective treatment as well. Exclusion criteria were: (1) case series or comparative non-randomized trials; (2) written in languages other than English; (3) not dealing with the treatment of FAI syndrome. We further excluded all duplicate articles, articles from nonpeer reviewed journals or articles lacking access to the full text. Conference presentations, narrative reviews, editorials, and expert opinions were also excluded. Two investigators extracted relevant data independently. The following data were extracted from each study: demographics, study design and level of evidence, follow-up times, treatment groups, evaluation scores adopted, and overall clinical findings. Discrepancies between the two reviewers were resolved by discussion and consensus, and the results were reviewed by the senior investigators. The final list of the selected studies is presented in Table 1.

The quality of the randomized controlled trials (RCTs) included was assessed independently by two reviewers using the Cochrane Risk of Bias Assessment Tool. The risk of bias was assessed as a judgment (high, low, or unclear) for individual elements from seven domains, as detailed in Table 2.

## 3. Results

In the present review, data from 749 patients were retrieved: 418 right side (55.8%) and 392 males (52.3%). Furthermore, 532 (71.0%) cases were classified as CAM impingement, 47 (6.3%) as Pincer FAI, 90 (12%) as mixed FAI and 80 cases (10.7%) were not specified. The mean follow-up was 14 months, and the weighted mean age was 34.7 years.

In the arthroscopy cluster 372 (49.7%) patients were analyzed: 213 right side (56.5%) and 190 males (51.1%). Moreover, 263 (70.7%) cases were classified as CAM impingement, 23 (6.2%) as Pincer FAI, 46 (12.4%) as mixed FAI and 40 (10.7%) cases were not specified. The weighted mean age was 34.7 years. The physiotherapy cluster accounts for 377 (50.3%) patients: 205 (54.4%) right side and 202 (53.6%) male gender; 269 (71.4%) cases were classified as CAM impingement, 24 (6.4%) as Pincer FAI, 44 (11.6%) as mixed FAI and 40 (10.6%) cases were not specified. The weighted mean age was 35.10 years.

From 749 patients enrolled in our review, only 620 (83.0%) completed the aimed follow-up: 55 (7.4%) were lost at follow-up in the arthroscopy cluster and 72 (9.6%) in the physiotherapy one. Furthermore, among all the studies included, 52 patients crossed over from conservative to surgical treatment, which represents 70% of non-surgical patients from the study conducted by Mansell et al. [12], 5% from Palmer et al. [15], 8% from Griffin et al. [11], and the 6% from Hunter et al. [14]

## 4. Reported Clinical Outcomes

### 4.1. International Hip Outcome Tool (iHOT-33)

The 33-item International Hip Outcome Tool (iHOT-33) is a questionnaire designed for self-administration which uses a visual analog scale format and can be provided to young active patients with pathologies affecting the hip.

Mansell et al. [12] reported a statistically significant improvement in iHOT-33 from baseline to 2 years in both groups, but the mean difference was not significant.

Similar results were reported by Griffin et al. [11] who documented an increase in the iHOT-33 score in both groups. Conversely to Mansell et al. [12], in the primary intention-to-treat analysis at 12 months, the iHOT-33 score was significantly higher in the hip arthroscopy group compared to the conservative group.

These results were consistent with the study by Palmer et al. [15] that confirmed a significantly higher iHOT-33 score in participants who received arthroscopic surgery compared to those who received the physiotherapy approach. Again, similar findings were found in the study by Hunter et al. [14] who showed a significant difference between the two groups at 12 months in favor of surgical treatment.

### 4.2. Hip Outcome Score of Daily Living (HOS-ADL) and Sports (HOS-Sports)

The Hip Outcome Score (HOS) activities of daily living (ADL) and sports subscales are self-reported outcomes with evidence of reliability and responsiveness for patients who are treated for arthroscopic hip surgery.

Mansell et al. [12] did not report any statistically significant difference between the surgery and physical therapy groups in HOS-ADL and HOS-sports at 6-month, 1-year and 2-year follow-ups. Conversely, Palmer et al. [15] found a significant difference of 10.0 points in HOS ADL in favor of the surgical group. Furthermore, 32% of patients treated in the physical therapy group and 51% of surgical patients reached the MCID (at least 9 points) in HOS-ADL, thus confirming the superior outcomes of the surgical group. Similarly, PASS (Patient acceptable symptomatic state, -defined as HOS-ADL ≥ 87 points-) was obtained in 19% of patients receiving physical therapy compared to 48% of patients receiving surgery.

### 4.3. EQ-5D 3L/5L and EQ-5D-5L-VAS

The EQ-5D 3L/5L and EQ-5D-5L-VAS are health surveys that can be used to compare improvement across different interventions by measuring changes in health-related quality of life over time.

Griffin et al. [11] found a statistically significant difference at 6 months in EQ-5D 3L/5L and EQ-5D 5L-VAS scores between the arthroscopy and conservative treatment group.

Hunter et al. [14] measured the baseline to 6-month and baseline to 12-month differences of these scores: comparing surgical and conservative groups, they reported significant improvement at 12 months relative to baseline in EQ-5D-5L, but not in EQ5D-VAS score, in favor of the surgical treatment. Finally, Palmer et al. [15] reported a statistically significant improvement at 6 months in the EQ-5D-3L index and EQ-5D-3L-VAS score in favor of arthroscopy.

### 4.4. Other Health-Related Scores

The global rating of change (GRC) is a score used to assess functional change over time in the clinical setting.

Mansell et al. [12] considered a GRC (Global Rating of Change) score to verify an eventual improvement of quality of life: 45.2% of patients in the arthroscopy group compared to 25.0% in the conservative treatment showed a GRC > 13, considered as the threshold for a satisfactory outcome. However, the relative risk of perceiving a statistically significant improvement was not different between the groups.

The Hip Disability and Osteoarthritis Outcome Score (HOOS) is a 40-item questionnaire used to assess patient-relevant outcomes in five separate subscales (pain, symptoms, activity of daily living, sport and recreation function and hip-related quality of life).

Hunter et al. [14] reported an improvement in the perceived quality of life in favor of the arthroscopy group compared to physiotherapy at 12 months by analyzing the Hip Disability and Osteoarthritis Outcome Score (HOOS) subscales: pain (*p* = 0.001), symptoms (*p* = 0.007), ADL (*p* = 0.000), sport (*p* = 0.003) and quality of life (*p* = 0.004). In all cases, better results were documented for the surgical group.

### 4.5. Delayed Gadolinium-Enhanced Magnetic Resonance Imaging (MRI) of Cartilage (dGEMRIC)

Hunter et al. [14] compared the dGEMRIC index between arthroscopy and physiotherapy groups at baseline and 12 months and showed no significant inter-group difference: although patients with symptomatic FAI experienced better outcomes after arthroscopic surgery, no imaging difference was detected to support these clinical findings.

## 5. Discussion

The present review highlighted the differences between surgical and conservative approaches in the treatment of femoro-acetabular impingement syndrome.

Formerly, Mok et al. [19], Dwyer et al. [20], and Gatz et al. [18] analyzed the three RCTs available until then. Our research added the latest RCT in literature (Hunter et al. [14]) and found the arthroscopic approach to be the preferred treatment for femoro-acetabular impingement syndrome in young and active patients. Our findings are comparable to results recently obtained in the work of Mahmoud et al. [21]

The subjective scores considered by the authors included iHOT-33, SF-12, EQ-5D-5L and HOS. iHot 33 is a clinical assessment tool for active patients which consists of symptoms, functional limitations, recreational activities, and sports and is, therefore, considered one of the main questionnaires to quantitatively evaluate patients’ symptoms [22]. The iHOT 33 tool demonstrated significantly better results for arthroscopic treatment in three out of the four studies included. Despite these notable results in favor of arthroscopy, potential biases should not be underestimated: Mansell et al. [12] enrolled military patients, thus introducing a potential bias in the generalizability of results. The study conducted by Mansell et al. [12] suffered from a high rate of crossover to surgery, lowering the statistical power of the results coming from the non-operative group. Moreover, both arthroscopy and physical therapy are predisposed to a performance bias since the administration of treatments could induce a placebo effect. To date, no blinded study has been conducted for the treatment of FAI: indeed, ethical considerations usually prevent from receiving approval to perform sham procedures, such as merely diagnostic arthroscopy or even skin incisions, which would be necessary to blind the patients. In an attempt to overcome this flaw, one study is currently ongoing and aims to compare arthroscopic treatment to sham surgery [23].

Furthermore, the role played by post-operative rehabilitation should not be underestimated [24]. The strength of the index hip has been recently demonstrated to be inferior in flexion, extension and adduction, up to 16 weeks following the arthroscopic procedure, compared to the contralateral healthy hip [25]. Therefore, effective postoperative rehabilitation could benefit from enhancing recovery after hip arthroscopy, thus speeding up the full healing of the patient [26]. The beneficial role of the surgical procedure was evident in most of the scores analyzed and these findings are in line with other similar studies on the topic [18]. Nonetheless, even if Griffin et al. [11] and Hunter et al. [14] adopted the same physiotherapy regimen based on an International Consensus, the large heterogeneity of the rehabilitation protocols adopted and the little evidence supporting the various programs, remarkably complicate a definitive conclusion in favor of arthroscopy. Looking at our results, three out of four high-quality evidence studies suggested the superiority of the arthroscopic treatment compared to the best conservative care, yet the optimal non-surgical treatment still lacks consensus. In everyday clinical practice, conservative treatment is usually proposed as a first-line approach although different regimens are proposed. Exercises focused on core strengthening are usually administered, even if their efficacy was proven only in small cohorts with different follow-up periods [27,28,29,30]. The rationale behind physiotherapy lies in relieving pain due to impingement by allowing the strengthening of the muscles and impeding unfavorable movements. However, the exact timing of the commonly administered exercises is not known, and duration displays large variability among the studies (Mansell et al. 12 sessions [12], Palmer et al. 8 sessions [15], Griffin et al. 10 sessions [11], Hunter et al. 6 sessions [14]). The trials conducted by Griffin et al. [11] and Hunter et al. [14] allowed intra-articular corticosteroids injection for pain relief in the non-operative group, which may have garbled the outcomes.

Many authors suggested a possible association between FAI syndrome and idiopathic hip osteoarthritis [31,32,33]: although hip arthroscopy seemed to provide superior functional results and better pain control, there are insufficient data to support the preventive role of surgery [34]. In fact, there is limited evidence on the long-term outcomes of hip arthroscopy in terms of OA progression: surgery might be not able to delay joint degeneration and relapse of symptoms compared to conservative treatment. Any surgical procedure is indeed able to impair the joint environment, so long-term evaluation is needed to understand the real risk/benefit ratio of hip arthroscopy over time; however, larger long-term studies are usually burdensome and their prohibitive costs will most likely affect the future evidence available. Furthermore, when considering such surgical procedures, one should not neglect the possible surgery-related complications: although in the cohort of patients analyzed (total number = 395), just two had notable complications (one fracture and one septic arthritis), previous studies found an adverse event rate following hip arthroscopy in up to 5% of patients [35].

Based on these findings, the optimal treatment for FAI remains uncertain.

Although we included only randomized controlled trials, several methodological limitations must be acknowledged: first, the small number of papers selected prevents the assessment of a definitive conclusion on the best standard of care for the FAI syndrome. The number of patients studied is still too small and not representative of the real incidence of the disease in the general population, estimated to be up to 17% of patients with groin pain [26]. Furthermore, FAI includes a wide spectrum of anatomical morphologies, requiring a tailored surgical approach. In the present analysis, no stratification was made based on the different subtypes of FAI and different surgical procedures performed. Furthermore, when considering physical therapy, we need to consider the compliance of the patients, which is must higher in the context of clinical trials compared to the real-world setting, where physiotherapy regimens are often discontinued due to working or social habits of patients; therefore, in real life, the outcomes following conservative treatment might be inferior to those reported in the RCTs.

## 6. Conclusions

Femoro-acetabular impingement syndrome is a common cause of pain and groin dysfunction in young active adults. Both arthroscopic treatment and a proper regimen of physical therapy are effective for pain relief and restoring functional status. However, the surgical approach seems to offer superior short-term results when compared to conservative care only. Further evaluations are needed to clarify whether surgery might prevail even at middle to long-term follow-up.

## Figures and Tables

**Table 1:** Synopsis of the main features of the RCTs included in the systematic review.

Study	Study Design	Treatment Groups	Outcome Measures	Follow-Up	Rehabilitation Program	Main Results	Comments on Results
Griffin et al. [11]	RCT	171 surgical and 177 PT	iHOT-33 EuroQol EQ-5D-5L SF-12	12mo	6 to 10 sessions over 12 to 24 weeks with physiotherapist personalized hip therapy with an assessment of pain, function, and range of hip motion; patient education; an exercise program that has the key features of individualization, progression, and supervision; help with pain relief, which could include one X-ray or ultrasound-guided intra-articular steroid injection	At 12 mo follow-up, there was a mean adjusted difference of 6·8 points in the iHOT-33 score between groups, in favor of hip arthroscopy. This is a statistically significant difference that also exceeded the minimum clinically important difference for iHOT-33.	Hip arthroscopy is more clinically effective than best conservative care
Mansell et al. [12]	RCT	38 surgical and 40 PT	HOS iHOT-33 GRC	24mo	12 sessions over 6 weeks with joint mobilizations, mobilization with motion, therapeutic exercise, soft tissue mobility, stretching, motor control exercises and home exercise program.	There was no significant difference between the surgery and no surgery groups at any time point out to 2 years on the HOS ADL and sport subscales or the iHOT-33. There was a statistically significant improvement from baseline to 1 and 2 years on the HOS ADL subscale and the iHOT-33 in the surgery group only.	Despite improvements over time, no meaningful change was perceived by most patients. A high rate of crossover to the surgery group affected the power of the study and prevents us from making definitive conclusions.
Palmer et al. [15]	RCT	112 surgical and 110 PT	HOS ADL HOS sport NAHS HAGOS OHS iHOT-33 EQ-5D-3L PainDETECT HADS	8mo	Up to 8 physiotherapy sessions over 5 mo with physiotherapist personalized hip therapy, with emphasis on improving core stability and movement control.	The mean HOS ADL in the arthroscopic surgery group was 10.0 points (95% confidence interval 6.4 to 13.6, *p* = 0.001) higher than in the physiotherapy program group at 8mo follow-up.	Patients with FAI syndrome experience a greater improvement in symptoms with arthroscopic hip surgery than with physiotherapy and activity modification at 8mo follow-up.
Hunter et al. [14]	RCT	49 surgical and 50 PT	dGEMRIC score HOAMS iHOT-33 HOOS SF-12 GIS Modified UCLA	12mo	6 PT sessions over 12 weeks. If needed 4 more PT sessions were added between 12 weeks and 6 months. 1. An individualized and progressive exercise program supervised by a physiotherapist. 2. Education about the condition and its Management. 3. Advice regarding pain relief which could include referral to the participants’ General Practitioner or ultrasound-guided intra-articular steroid injection.	The primary outcome of hip cartilage metabolism dGEMRIC showed no statistically significant difference Between PHT and arthroscopic hip surgery at 12 months follow-up. the range of secondary outcomes demonstrated statistically and clinically important improvements with significance between group differences favoring surgery.	This trial adds new information that shows the patient reported benefits of surgery are not explained by nor linked to better hip cartilage metabolism at 12 months.

**Table 2:** Cochrane Risk of Bias assessment for all the included studies. + Low risk of bias; − High risk of bias.

	Selection Bias Random Sequence Generation	Selection Bias Allocation Concealment	Reporting Bias Selective Reporting	Performance Bias Blinding (Participants and Personnel)	Detection Bias Blinding (Outcome Assessment)	Attrition Bias Incomplete Outcome Data	Other Bias
Griffin et al. [11]	+	+	+	−	+	+	+
Mansell et al. [12]	+	+	−	−	−	−	−
Palmer et al. [15]	+	+	+	−	+	+	+
Hunter et al. [14]	+	−	+	−	+	−	−
